# Body Image and Nutritional Status Among Adolescents and Adults

**DOI:** 10.3390/nu17233655

**Published:** 2025-11-22

**Authors:** Emanuela Gualdi-Russo, Luciana Zaccagni

**Affiliations:** 1 Department of Neuroscience and Rehabilitation, Faculty of Medicine, Pharmacy and Prevention, University of Ferrara, 44121 Ferrara, Italy; luciana.zaccagni@unife.it; 2 Center for Exercise Science and Sports, University of Ferrara, 44123 Ferrara, Italy

## 1. Introduction

Body image perception is a composite construct that includes feelings, evaluations, and attitudes concerning the body. This perception may be independent of the body’s actual appearance, and is strongly influenced by others’ judgments. Body image dissatisfaction may arise from negative thoughts and feelings about one’s own body [1].

Disturbances to body image, including dissatisfaction with and misperception of one’s body, may be associated with eating disorders and unhealthy behaviors aimed at weight control [2].

The idealization of thinness is widely observed in Western societies, particularly among women and in those of increasingly younger ages. This can lead to dissatisfaction with one’s body and low self-esteem, resulting in a distorted perception of body image [3,4].

Distorted body image is common and can cause anxiety regarding one’s appearance, as well as depressive disorders and harmful eating behaviors, especially among adolescents and young adults. Furthermore, body dissatisfaction resulting from discrepancies between perceived and ideal body image can negatively affect individuals in terms of both physical and mental health.

This Special Issue of *Nutrients* collates twelve original research articles that discuss various aspects of body perception and eating disorders. The main aim of this Special Issue is to present an overview of the recent advances in this field, encompassing risk factors for eating disorders as well as the effects of physical activity on body image, from dissatisfaction with weight and body image to anorexia nervosa. Furthermore, it aims to explore differences in gender and age across a diverse range of contexts.

## 2. Eating Disorders, Risk Factors, and Assessment Tools

An article by Bozzola et al., forming contribution 1, shows that the experience of multiple adverse events, particularly during childhood and adolescence, can significantly increase the likelihood of an individual developing an eating disorder. Based on a sample of patients under 18 years of age hospitalized for anorexia, the authors hypothesized that considering the simultaneous presence of multiple potential risk factors, rather than a single risk factor, is crucial to understanding the severity of anorexia nervosa.

Especially in sports such as artistic and rhythmic gymnastics, in which success is associated with maintaining a lean body to meet esthetic and performance demands, participation is associated with a heightened risk of eating disorders. In this context, Donti et al. (contribution 2) demonstrate that adolescent artistic gymnasts exhibit symptoms of eating disorders and have limited knowledge of the key aspects of Relative Energy Deficiency in Sport (RED-S). Notably, a higher prevalence of eating disorders has been found among elite gymnasts compared to those at lower competitive levels. This suggests that reduced pressure on performance and appearance is associated with fewer eating disorders.

The incidence of anorexia nervosa was observed to have increased among children and adolescents during the pandemic. A study by Dylag et al. [contribution 3], conducted on a large sample of Polish patients aged 10 to 18, demonstrated the significant impact of the COVID-19 pandemic on the clinical course and hospitalization patterns of pediatric patients with anorexia nervosa. The results suggest that the pandemic may have worsened the disease’s severity and resulted in alterations to treatment approaches, underscoring the need for increased awareness, early intervention, and targeted support strategies to ameliorate the impact of future pandemics on the incidence of eating disorders.

A diet rich in plant-based nutrients such as the Mediterranean diet has been shown to reduce plasma levels of pro-inflammatory cytokines, thus counteracting the biological mechanisms involved in the development of depression. In this context, Menghi et al. (contribution 4) found that the girls they examined exhibited higher levels of depression, anxiety, neuroticism, and food pickiness than the boys. They also observed that greater sensitivity to bitterness and astringency predicted higher levels of depressive symptoms in girls exclusively. This suggests that gender modulates the relationship between depressive symptoms, sensory perception, and eating habits in healthy adolescents.

The symptoms of eating disorders are complex and heterogeneous, making their clinical evaluation challenging. Despite the many validated assessment tools that have been developed, several demonstrated limited sensitivity to change, an inadequate coverage of associated factors, and excessive reliance on indicators such as BMI. Addressing this drawback, Golan et al. (contribution 5) examined the psychometric properties of the CONTASI-ED tool, demonstrating its strong reliability and that it has a high correlation with EAT-26. The scores acquired decreased significantly over the course of treatment, confirming the tool’s sensitivity to changes in symptoms.

Furthermore, Gallagher et al. (contribution 6) investigated the Eating Disorders-Specific Nutrition-Focused Physical Examination (ED-NFPE) tool for use by registered dietitian nutritionists across different levels of emergency department care. Developed through expert consensus, this tool assesses nutrition-related complications in individuals with eating disorders. The results demonstrate that the ED-NFPE tool can enhance nutritional assessments and improve the overall patient experience.

## 3. Dissatisfaction with Body Image Perception and Weight

Girls, in particular, tend to be dissatisfied with their body weight. Fondell et al. (contribution 7) compared girls who wished to lose weight with those who intended to maintain their current weight in a nationally representative U.S. sample, examining eating habits, physical activity, vaping, alcohol consumption, sleep patterns, and screen time. Adolescent girls with the intention to lose weight exhibited various unhealthy behaviors, including skipping breakfast, increased screen time, and sleeping less. They also reported higher alcohol consumption and more vaping. Although weight loss intentions were more common among obese girls, they were also observed to have considerable prevalence among normal-weight girls. The authors suggested that girls would benefit from interventions targeting sleep, screen time, diet, physical activity, and substance use.

Negative body image perception is a hallmark symptom of anorexia nervosa. Affected individuals often demonstrate significantly distorted body size perception and high dissatisfaction with their body, including its size, shape, and appearance. A study by Scarpina et al. (contribution 8) revealed that muscle mass and vitamin B6 concentrations are associated with the psychological manifestations of body image concerns in individuals with anorexia nervosa, particularly in the early stages of the disorder.

Adolescents generally tend to be dissatisfied with their bodies due to discrepancies between their current body image and prevalent beauty ideals. Through a longitudinal study of early adolescents, Gualdi-Russo et al. (contribution 9) found that perceptions of body image remained relatively stable over the three-year study period. Dissatisfaction was observed at similar rates in both genders, although different esthetic ideals were already noticeable even in early adolescence. Self-perception of weight status was not entirely consistent, with underweight adolescents tending to overestimate it and overweight/obese adolescents tending to underestimate it. Given increasing body dissatisfaction and the increasing misinterpretations of weight status, the promotion of a healthy lifestyle and a positive body image in schools is an advisable endeavor.

Relationships between body image and sociodemographic, morphological, and behavioral variables were examined by Knapik et al. (contribution 10) in a sample of adult women. The results indicate that women generally tend to be critical of their bodies. Excessive BMI negatively influences scores on the Body Esteem Scale, while BMI itself is less severely affected by sociodemographic variables. Engagement in physical activity had a particularly notable correlation with the “physical condition” domain of the Body Esteem Scale in young women.

Given that positive body image is linked to improved physical and mental well-being, Kriaučionienė et al. (contribution 11) examined the associations between body appreciation, body weight, lifestyle factors, and subjective health among undergraduate students. Their study highlighted strong links between body appreciation, healthy weight, positive lifestyle behaviors, and self-perceived health. The promotion of healthier habits may therefore foster an increased sense of body appreciation.

While numerous studies have explored body image and self-esteem in females, only a few have examined these aspects in adult men. McNeill’s study (contribution 12) found that men are generally less influenced by celebrities or fashion trends in the development of their body image ideals and are largely satisfied with their appearance. The author found no significant influence of age on men’s body image; instead, body image satisfaction appears to be shaped by other aspects of life satisfaction, such as relationship status and engagement in sports.

## 4. Conclusions

Altogether, the studies presented in this Special Issue emphasize the importance of body image perception and nutritional status for both physical and mental health. Furthermore, the availability of validated tools can substantially assist in clinical and nutritional assessments.

In conclusion, ongoing research on body image and weight perceptions, as well as the related eating disorders, continues to elucidate human variability and the factors that influence health maintenance. Nevertheless, further clinical, translational, nutritional, and anthropometric research is needed to address the many questions that remain unanswered.
